# Microwave Assisted Synthesis of Some New Heterocyclic Spiro-Derivatives with Potential Antimicrobial and Antioxidant Activity

**DOI:** 10.3390/molecules15128827

**Published:** 2010-12-03

**Authors:** Mohamed Mohamed Youssef, Mahmoud Ahmed Amin

**Affiliations:** 1 Chemistry Department, Faculty of Science, Cairo University, Egypt; 2 Chemistry Department, Faculty of Science, Suez Canal University, Egypt

**Keywords:** microwave, spiro-compounds, heterocycles, spiro-benzoxazoleisoquinoline, spiro-benzimidazoleisoquinoline, spiro-isoquinolinetriazole, spiro-isoquinolinepyrazole, spiro-isoquinolinepyrimidine, antimicrobial activity, antioxidant activity

## Abstract

Homophthalic anhydride reacts with different aromatic amines to produce *N*-substituted homophthalimides. Bromination of the latter produces 4,4-dibromo-homophthalimide derivatives that can be used as precursors for spiro-derivatives. The dibromo derivatives react with different binucleophilic reagents to produce several spiro-isoquinoline derivatives. Reaction of the dibromo derivatives with malononitrile produces dicyanomethylene derivatives which react with different binucleophiles to produce new spiro-derivatives. Structures of the newly synthesized compounds are proved using spectroscopic methods such as IR, ^1^H-NMR and ^13^C-NMR. The newly synthesized compounds were tested for their antimicrobial and antioxidant activities, showing weak or no antimicrobial activity. On the other hand select compounds showed promising antioxidant activities.

## 1. Introduction

Spiro-compounds form a group of generally less investigated compounds. However, recently growing efforts have been made to synthesize and characterize these compounds. Many spiro-compounds possess very promising biological activities as anticancer agents [1,2], antibacterial agents [3,4], anticonvulsant agents [5,6,7], anti-tuberculosis agents [8], anti-Alzheimer’s agents [9], pain-relief agents [10,11], anti-dermatitis agents [12] and antimicrobial agents [13,14]. In addition to their medical uses, some spiro-compounds have found other uses in the agricultural and industrial fields. For example, they are used as antifungal agents [15], pesticides [16], laser dyes [17] and electroluminescent devices [18]. Spiro compounds have also been recently used as antioxidants [19,20]. We were prompted by these findings to try to synthesize new spiro-compounds with potential antimicrobial or antioxidant activities.

The microwave technique has several advantages over traditional methods of synthesis. Reduced reaction times [21,22,23,24], less effects on the environment and better reaction yields are some of the common advantages of using microwaves. In the present research project, we used both the microwave technique as well as conventional methods to prepare some spiro-compounds with expected biological activity.

## 2. Results and Discussion

### 2.1. Chemistry

**Scheme 1:**
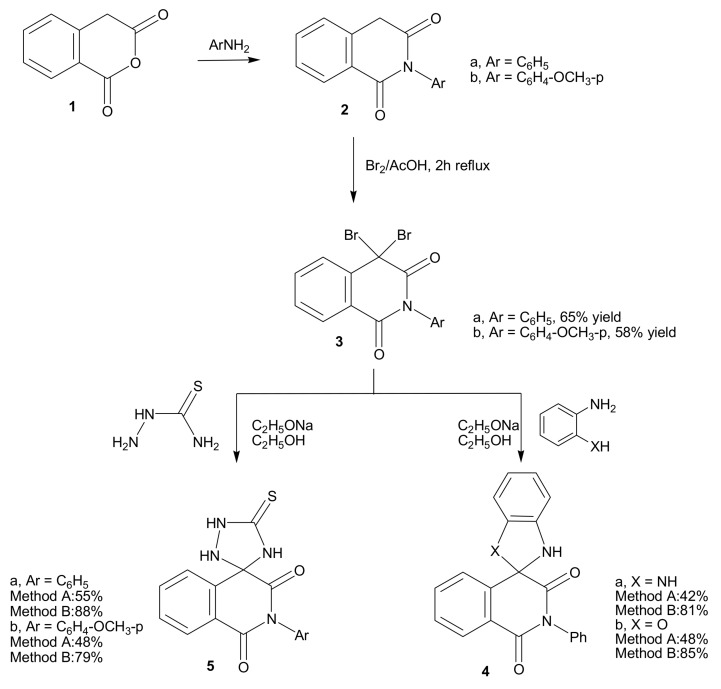
Reactions of dibromohomophthalimides with binucleophilic reagents; synthesis of **4** and **5**.

Homophthalic anhydride (**1**) was reacted with aromatic amines, namely aniline and *p*-anisidine, to afford *N*-arylhomophthalimide derivatives **2a,b**, respectively, which were used as precursors for preparing new spiro-isoquinolines (Scheme 1). Compounds **2a,b**, having an active methylene group, reacted with two equivalents of bromine in acetic acid to produce 2-aryl-4,4-dibromoisoquinoline-1,3-(2*H*,4*H*)dione derivatives **3a,b.** The mass spectrum of compound **3a** displayed the expected molecular ion peaks at *m/z* 394 (3.2%), 395 (6.7%), 396 (3.0%). Compound **3b** gave molecular ion peaks at 424 (7.3%), 425 (15.1%), 426 (6.8%). 

Compound **3a** underwent direct cyclocondensation when treated with each of *o*-phenylenediamine or *o*-aminophenol to produce 2'-phenyl-1,3-dihydro-1'*H*-spiro-[benzimidazole-2,4'-isoquinoline]-1',3' (2'H)-dione (**4a**) and 2'-phenyl-1'*H*,3*H*-spiro[1,3-benzoxazole-2,4'-isoquinoline]-1',3'(2'*H*)-dione (**4b**), respectively. Compounds **4a** and **4b** are new ring systems. Synthesis of compounds **4a,b** was carried out under two different reaction conditions, namely by conventional heating and using microwave irradiation conditions. Thus, when the reaction was carried out in a refluxing ethanolic sodium ethoxide solution for 5 h under TLC monitoring, the product **4a** was obtained in 42% yield, while **4b** was obtained in 48% yield. However, when the same reaction was carried out by heating at 140 °C in a microwave oven for 15 min, the yields of **4a** and **4b** were 81% and 85%, respectively. It was then concluded that using microwave as a source of heat not only improves the reaction yield, but also significantly reduces the reaction time. The IR and ^1^H-NMR spectra of compounds **4a** and **4b** agreed with the proposed structures. Their mass spectra showed the molecular ion peaks at *m/z* 341 (12.3%) and 342 (8.6%), respectively.

Similarly, compounds **3a,b** reacted with thiosemicarbazide under both conventional and microwave reaction conditions and produced 2-phenyl-5'-thioxo-1*H*-spiro[isoquinoline-4,3'-[1,2,4]triazolidine]-1,3(2*H*)-dione (**5a**) and 2-(4-methoxyphenyl)-5'-thioxo-1*H*-spiro-[isoquinoline-4,3'-[1,2,4]-triazol-idine]-1,3(2*H*)-dione (**5b**), respectively. Compounds **5** also have a new ring system. Again, comparing the reaction times and overall yields for both traditional and microwave methods showed that traditional methods took 4 h, to give 55% and 48% yields of **5a** and **5b**, respectively, while the microwave method needed only 15 min to give 88% and 79% yields of **5a** and **5b**, respectively. The comparison again proves the advantages of using the microwave technique. Analytical and spectral data of **5a** and **5b** were in agreement with the proposed structures.

**Scheme 2:**
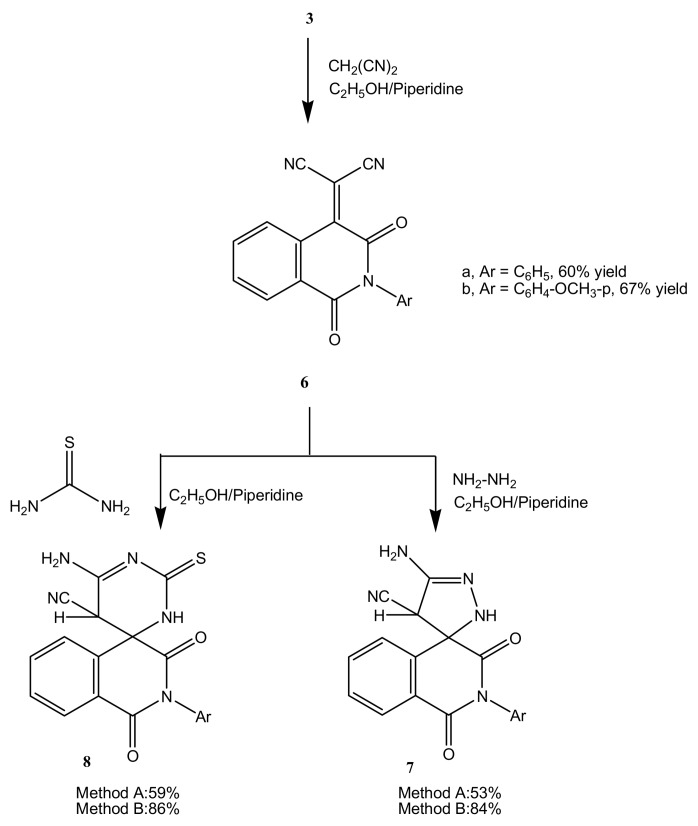
Reactions of isoquinolinylidenemalononitriles with hydrazine hydrate and thiourea; synthesis of **7** and **8.**

On the other hand, compounds **3a,b** reacted with malononitrile, as an activated methylene compound, by refluxing under TLC monitoring in absolute ethanol containing a catalytic amount of piperidine for 3 h, to produce (1,3-dioxo-2-phenyl-2,3-dihydroisoquinolin-4(1*H*)-ylidene)-malononitrile (**6a**) and [2-(4-methoxyphenyl)-1,3-dioxo-2,3-dihydroisoquinolin-4(1*H*)-ylidene]-malononitrile (**6b**), respectively (Scheme 2). Attempts to carry out the same reaction under microwave conditions failed however, due to the formation of a complex mixture of products that could not be separated. Compounds **6a** and **6b** gave acceptable analytical and spectral data.

Compound **6a** could be used as a precursor for some spiro-isoquinolines containing five and six-membered heterocycles. Thus, compound **6a** reacted with each of hydrazine hydrate and thiourea to produce 5'-amino-1,3-dioxo-2-phenyl-2,2',3,4'-tetrahydro-1*H*-spiro[isoquinoline-4,3'-pyrazole]-4'-carbonitrile (**7**) and 6'-amino-1,3-dioxo-2-phenyl-2'-thioxo-2,2',3,5'-tetrahydro-1*H*,3'*H*-spiro-[isoquinoline-4,4'-pyrimidine]-5'-carbonitrile (**8**), respectively. Compound **8** has a new ring system. Synthesis of compounds **7** and **8** was performed under TLC monitoring in refluxing ethanol solution containing catalytic amounts of piperidine for 4 h and the yields were 53% and 59%, respectively. Carrying out the same reaction under microwave assisted conditions reduced the reaction time to 15 min and increased the yields to 84% and 86%, respectively, for **7** and **8**. Acceptable analytical data were obtained for the new compounds **7** and **8**.

### 2.2. Biological Evaluation

#### 2.2.1. Antimicrobial evaluation 

The newly synthesized heterocyclic compounds listed in Table 1 were tested for their antimicrobial activity against the following microorganisms: *Escherichia coli*, *Pseudomonas putida*, *Bacillus subtilis*, *Streptococcus lactis*, *Aspergillus niger*, *Penicillium sp.* and *Candida albicans*. The preliminary screening of the investigated compounds was performed using the filter paper disc-diffusion method. The most active compounds were **4a**, **5a**, **5b** and **8**, which were slightly inhibitory to the microorganisms**.** The rest of compounds showed no sensitivity at all to the tested organisms, and the results are summarized in Table 1.

#### 2.2.2. Anti-oxidant activity screening

The newly synthesized compounds were tested for anti-oxidant activity as reflected in the ability to inhibit lipid peroxidation in rat brain and kidney homogenates and rat erythrocyte hemolysis. The pro-oxidant activities of the aforementioned compounds were assessed by their effects on bleomycin-induced DNA damage. Table 2 shows the anti-oxidant assays by erythrocyte hemolysis, which reveals that compounds **7** and **8** manifested potent anti-oxidative activity in the lipid peroxidation assay and considerable inhibitory activity in the hemolysis assay. Table 3 shows the anti-oxidant assays by the ABTS method. Again, compounds **7** and **8** showed interesting anti-oxidant activity. Table 2 and Table 3 also show that compounds **4a** and **4b** have moderate anti-oxidant properties. All compounds have been tested on bleomycin-dependent DNA damage. The results indicate that some compounds, namely **4a**, **4b**, **7** and **8**, may have some protective activity towards DNA from the damage induced by bleomycin (**Table 4**).

## 3. Experimental 

### 3.1. General

Melting points were determined in open glass capillaries on a Gallenkamp melting point apparatus and are uncorrected. IR spectra (KBr discs) were recorded on a Shimadzu FTIR-8201PC spectrophotometer. ^1^H-NMR and ^13^C-NMR spectra were recorded on a Varian Mercury 300 MHz, and a Varian Gemini 200 MHz. spectrometers using TMS as an internal standard and DMSO-d_6_ as solvent. Chemical shifts were expressed as δ (ppm) units. Mass spectra were recorded at 70 eV on a Shimadzu GCMS-QP1000EX using an inlet type injector. All reactions were followed by TLC (silica gel, aluminum sheets 60 F254, Merck). The Microanalytical Center of Cairo University performed the microanalyses. Microwave reactions were performed with a Millstone Organic Synthesis Unit (MicroSYNTH with touch control terminal) with a continuous focused microwave power delivery system in a pressure glass vessel (10 mL) sealed with a septum under magnetic stirring. The temperature of the reaction mixture was monitored using a calibrated infrared temperature control under the reaction vessel, and control of the pressure was performed with a pressure sensor connected to the septum of the vessel.

#### 3.1.1. 2-aryl-4,4-dibromoisoquinoline-1,3-(2*H*,4*H*)dione derivatives **3a,b**

A solution of either of **2a** (2.37 g, 0.01 mol) or **2b** (2.76 g, 0.01 mol) in glacial acetic acid (20 mL) was heated under reflux with bromine (1.1 mL, 3.0 g, 0.02 mole) for 2 h. After cooling, the reaction mixture was poured onto ice-water and the solid that precipitated was filtered off, dried and crystallized from the proper solvent.

*4,4-Dibromo-2-phenylisoquinoline-1,3-(2H,4H)dione* (**3a**): crystallized from ethanol in 65% yield as white crystals, m.p. 236-237 °C; ^1^H-NMR: 7.10-8.30 (m, 9H, Ar-H); ^13^C-NMR: 70.2 (sp^3^ C-4), 120.5, 123.2, 124.3, 126.1, 127.4, 130.0, 131.5, 134.6, 135.2, 136.4 (aromatic C), 158.4, 167.6 (2 CO); IR*ν*: 3066 cm^−1^ (aromatic CH), 1645 (broad, 2C=O), 1605, 1500 (aromatic C=C); MS: M^+^ *m/z* 394 (3.2%), 395 (6.7%), 396 (3.0%); Anal. Calcd. for C_15_H_9_Br_2_NO_2_ (395.04): C (45.60%), H (2.30%), Br (40.45%), N (3.55%); Found: C (45.3%), H (2.4%), Br (45.5%), N (3.7%).

*4,4-Dibromo-2-(4-methoxyphenyl)isoquinoline-1,3-(2H,4H)dione* (**3b**): crystallized from dilute dioxane in 58% yield as white crystals, m.p. 255-256 °C; ^1^H-NMR: 3.60 (s, 3H, OCH_3_), δ 6.80-7.90 ppm (m, 8H, Ar-H); ^13^C-NMR: 57.1 (OCH_3_), 71.0 (sp3 C-4), 115.3, 118.2, 124.2, 126.0, 127.2, 130.3, 131.6, 134.4, 135.1, 152.2 (aromatic C), 155.4, 163.6 (2 CO); IR*ν*: 3060 cm^−1^ (aromatic CH), 1640 (broad, 2C=O), 1605, 1500 (aromatic C=C); MS: M^+^ *m/z* 424 (7.3%), 425 (15.1%), 426 (6.8%); Anal. Calcd. for C_16_H_11_Br_2_NO_3_ (425.07): C (45.21%), H (2.61%), Br (37.60%), N (3.30%); Found: C (45.1%), H (2.5%), Br (45.0%), N (3.6%).

#### 3.1.2. Cyclocondensation of **3a** with *o*-phenylenediamine and *o*-aminophenol; formation of **4a**,**b**.

*Method A:* Compound **3a** (3.95 g, 0.01 mole) was heated under reflux with either of *o*-phenylenediamine (1.08 g, 0.01 mol) or *o*-aminophenol (1.09 g, 0.01 mol) in ethanolic sodium ethoxide solution [prepared by dissolving metallic sodium (0.27 g, 0.012 mol) in absolute ethanol (25 mL)] for 5 h, under TLC monitoring. The reaction mixture was then cooled, acidified with few drops of conc. hydrochloric acid and the solid that precipitated was filtered at the pump and crystallized from the appropriate solvent.

*Method B*: The same reactants of method A were heated in microwave at 500 W and 140 °C for 15 min. The reaction mixture was treated in a similar manner to method A to obtain compounds **4a,b**.

*2'-Phenyl-1,3-dihydro-1'H-spiro[benzimidazole-2,4'-isoquinoline]-1',3'(2'H)-dione* (**4a**): Crystallized from dimethylformamide as grey crystals, m.p. 218-219 °C, in 42% yield (Method A) or 81% yield (Method B); ^1^H-NMR: 3.80 (s, 2H, 2NH, D_2_O exchangeable), 6.40-7.70 (m, 13H, Ar-H); ^13^C-NMR: 83.4 (sp^3^ spiro C), 113.0, 115.9, 120.2, 122.9, 127.3, 127.8, 128.3, 128.9, 131.2, 133.7, 135.1, 135.9, 136.8 (aromatic C), 155.4, 163.6 (2 CO); IR*ν*: 3180 cm^−1^ (broad, NH), 3065 (aromatic CH), 1645,1655 (2C=O), 1605, 1500 (aromatic C=C); MS: M^+^ *m/z* 341 (12.3%); Anal. Calcd. for C_21_H_15_N_3_O_2_ (341.36): C (73.89%), H (4.43%), N (12.31%); Found: C (74.1%), H (4.4%), N (12.1%).

*2'-Phenyl-1'H,3H-spiro[1,3-benzoxazole-2,4'-isoquinoline]-1',3'(2'H)-dione* (**4b**): Crystallized from dioxane as white crystals, m.p. 237-238 °C, in 48% yield (Method A) and 85% yield (Method B); ^1^H-NMR: 4.30 (s, 1H, NH, D_2_O exchangeable), 6.60-7.80 (m, 13H, Ar-H); ^13^C-NMR: 83.4 (sp^3^ spiro C), 116.0, 117.9, 118.2, 121.2, 124.9, 125.8, 127.5, 128.1, 128.5, 129.9, 132.1, 134.5, 135.4, 136.3, 138.8, 144.9 (aromatic C), 160.0, 168.9 (2 CO); IR*ν*: 3210 cm^−1^ (NH), 3065 (aromatic CH), 1655,1670 (2C=O), 1605, 1500 (aromatic C=C); MS: M^+^ *m/z* 342 (8.6%); Anal. Calcd. for C_21_H_14_N_2_O_3_ (342.35): C (73.68%), H (4.12%), N (8.18%); Found: C (73.3%), H (4.2%), N (7.9%).

#### 3.1.3. Cyclocondensation of **3a,b** with thiosemicarbazide; formation of **5a,b**

*Method A:* Each of *c*ompounds **3a** (3.95 g, 0.01 mol) and **3b** (4.25 g, 0.01 mol) was heated under reflux with thiosemicarbazide (0.91 g, 0.01 mol) in ethanolic sodium ethoxide solution [prepared by dissolving metallic sodium (0.27 g, 0.012 mol), in absolute ethanol (25 mL)] for 4 h, under TLC monitoring. The reaction mixture was then cooled, acidified with few drops of conc. hydrochloric acid and the solid that precipitated was filtered at the pump and crystallized from the appropriate solvent.

*Method B:* The same reactants of method A were heated in microwave at 500 W and 140 °C for 15 min. The reaction mixture was treated in a similar manner to method A to give compounds **5a,b**.

*2-Phenyl-5'-thioxo-1H-spiro[isoquinoline-4,3'-[1,2,4]triazolidine]-1,3(2H)-dione* (**5a**): Crystallized from ethanol as white crystals, m.p. 188-189 °C, in 55% yield (Method A) and 88% yield (Method B); ^1^H-NMR: 2.10 (s,1H, NH, D_2_O exchangeable), 2.30 (s,1H, NH, D_2_O exchangeable), 2.40 (s, 1H, NH, D_2_O exchangeable), 7.10-8.30 (m, 9H, Ar-H); ^13^C-NMR: 77.7 (sp^3^ spiro C), 121.1, 123.4, 127.6, 128.4, 128.8, 129.8, 132.1, 134.0, 135.2, 136.8 (aromatic C), 154.9 (CS), 159.5, 166.9 (2 CO); IR*ν*: 3220, 3185, 3150 cm^−1^ (NH), 3060 (aromatic CH), 1645,1670 (2C=O), 1600, 1490 (aromatic C=C); MS (70 eV): M^+^ *m/z* 324 (11.3%); Anal. Calcd. for C_16_H_12_N_4_O_2_S (324.36): C (59.25%), H (3.73%), N (17.27%), S (9.89%); Found: C (59.5%), H (3.9%), N (16.9%), S (10.0%).

*2-(4-Methoxyphenyl)-5'-thioxo-1H-spiro[isoquinoline-4,3'-[1,2,4]-triazolidine]-1,3(2H)-dione* (**5b**): Crystallized from dilute dioxane as white crystals, m.p. 202-203 °C, in 48% yield (Method A) and 79% yield (Method B); ^1^H-NMR: 2.10 (s, 1H, NH, D_2_O exchangeable), 2.30 (s, 1H, NH, D_2_O exchangeable), 2.40 (s, 1H, NH, D_2_O exchangeable), 3.70 (s, 3H, OCH_3_), 7.10-8.30 (m, 9H, Ar-H); IR *ν*: 3220, 3185, 3150 cm^−1^ (NH), 3060 (aromatic CH), 1640, 1660 (2C=O), 1600, 1490 (aromatic C=C); MS: M^+^ *m/z* 354 (13.8%). Anal. Calcd. for C_17_H_14_N_4_O_3_S (354.38): C (57.62%), H (3.98%), N (15.81%), S (9.05%); Found: C (57.3%), H (3.9%), N (16.1%), S (9.3%).

#### 3.1.4. Reaction of **3a,b** with malononitrile: formation of **6a,b**

To a solution of each of compounds **3a** (3.95 g, 0.01 mol) and **3b** (4.25 g, 0.01 mol) in absolute ethanol (30 mL) containing a catalytic amount of piperidine was added malononitrile (0.66 g, 0.01 mol). The reaction mixture was heated under reflux for 3 h, under TLC monitoring, then cooled and poured onto ice-cold water. The solid product that separated was filtered off, dried and crystallized from ethanol.

*(1,3-Dioxo-2-phenyl-2,3-dihydroisoquinolin-4(1H)-ylidene)-malononitrile* (**6a**): Obtained in 60% yield as pale yellow crystals, m.p. 224-225 °C; ^1^H-NMR: 7.00-8.10 (m, 9H, Ar-H); ^13^C-NMR: 83.1 (ethylenic C), 112.0 (CN), 122.4, 124.7, 127.1, 127.8, 128.2, 128.6, 129.9, 131.0, 133.1, 133.9 (aromatic C), 147.3 (C3), 158.1, 163.0 (2 CO); IR*ν*: 3060 cm^−1^ (aromatic CH), 2185 (CN), 1710, 1660 (2C=O), 1600, 1490 (aromatic C=C); MS: M^+^ *m/z* 299 (28.2%); Anal. Calcd. for C_18_H_9_N_3_O_2_ (299.28): C (72.24%), H (3.03%), N (14.04%); Found: C (72.0%), H (3.3%), N (14.3%).

*[2-(4-Methoxyphenyl)-1,3-dioxo-2,3-dihydroisoquinolin-4(1H)-ylidene]malononitrile* (**6b**): Obtained as yellow crystals in 67% yield, m.p. 241-242 °C; ^1^H-NMR: 3.70 (s, 3H, OCH_3_), 6.70-8.00 (m, 8H, Ar-H); ^13^C-NMR: 57.3 (OCH_3_), 83.0 (ethylenic C), 113.1 (CN), 115.2, 124.1, 126.4, 127.2, 127.9, 128.3, 129.5, 131.0, 133.1, 144.2 (aromatic C), 146.9 (C3), 158.4, 163.2 (2 CO; IR*ν*: 3060 cm^−1^ (aromatic CH), 2195 (CN), 1710, 1670 (2C=O), 1600, 1490 (aromatic C=C); MS: M^+^ *m/z* 329 (24.8%); Anal. Calcd. for C_19_H_11_N_3_O_3_ (329.31): C (69.30%), H (3.37%), N (12.76%); Found: C (69.5%), H (3.3%), N (12.4%).

#### 3.1.5. *5'-Amino-1,3-dioxo-2-phenyl-2,2',3,4'-tetrahydro-1H-spiro-[isoquinoline-4,3'-pyrazole]-4'-carbonitrile* (**7**).

*Method A*: A solution of **6a** (2.99 g, 0.01 mol) in absolute ethanol (30 mL) containing a catalytic amount of piperidine was heated under reflux with hydrazine hydrate (1.1 mL, 0.02 mol) for 4 h under TLC monitoring. The reaction mixture was then cooled, poured onto ice-cold water. The solid that separated was filtered off, dried and crystallized from dilute dimethylformamide.

*Method B*: The same reactants of method A were heated in microwave at 500 W and 140 °C for 15 min. The reaction mixture was treated in a similar manner to method A to obtain compound **7**.

Compound **7** was obtained as white crystals, m.p. 254-255 °C, in 53% yield (Method A) and 84% yield (Method B); ^1^H-NMR: 2.10 (s, 2H, NH_2_, D_2_O exchangeable), 3.50 (s,1H, pyrazole H4), 6.10 (s, 1H, NH, D_2_O exchangeable) 6.90-8.10 (m, 9H, Ar-H); ^13^C-NMR: 44.1 (pyrazole C4), 55.3 (sp^3^ spiro C), 116.1 (CN), 120.2, 123.1, 126.9, 128.2, 128.9, 129.1, 129.7, 132.0, 135.1, 144.4 (aromatic C), 150.9 (pyrazole C3), 155.3, 169.2 (2 CO); IR *ν*: 3340, 3315, 3260 cm^−1^ (NH_2_ and NH), 3080 (aromatic CH), 2180 (CN), 1670,1640 (2C=O), 1600, 1490 (aromatic C=C); MS: M^+^ *m/z* 331 (7.8%); Anal. Calcd. for C_18_H_13_N_5_O_2_ (331.33): C (65.25%), H (3.95%), N (21.14%); Found: C (65.5%), H (4.1%), N (21.5%).

#### 3.1.6. *6'-Amino-1,3-dioxo-2-phenyl-2'-thioxo-2,2',3,5'-tetrahydro-1H,3'H-spiro[isoquinoline-4,4'-pyrimidine]-5'-carbonitrile* (**8**)

*Method A*: A solution of **6a** (2.99 g, 0.01 mol) in absolute ethanol (30 mL) containing a catalytic amount of piperidine was heated under reflux with thiourea (0.76 g, 0.01 mol) for 4 h under TLC monitoring. The reaction mixture was then cooled, poured onto ice-cold water. The solid that separated was filtered off, dried and crystallized from dilute dimethylformamide. 

*Method B*: The same reactants of method A were heated in microwave at 500 W and 140 °C for 15 min. The reaction mixture was treated in a similar manner to method A to obtain compound **8**.

Compound **8** was obtained as white crystals, m.p. 243-244°C, in 59% yield (Method A) and 86% yield (Method B); ^1^H-NMR: 2.10 (s, 2H, NH_2_, D_2_O exchangeable), 2.70 (s, 1H, NH, D2O exchangeable), 3.30 (s, 1H, pyrimidine H5), 6.90-7.90 (m, 9H, Ar-H); ^13^C-NMR: 39.3 (pyrimidine C5), 54.9 (sp^3^ spiro C), 118.4 (CN), 121.1, 124.6, 126.3, 128.8, 129.4, 129.8, 130.76, 132.7, 135.9, 143.6 (aromatic C), 150.9 (pyrimidine C4), 158.1 (CS), 161.3, 169.2 (2 CO); IR *ν*: 3300, 3285, 3180 cm^−1^ (NH_2_ and NH), 3070 (aromatic CH), 2150 (CN), 1670,1645 (2C=O), 1600, 1490 (aromatic C=C); MS: M^+^ *m/z* 375 (5.8%); Anal. Calcd. for C_19_H_13_N_5_O_2_S (375.40): C (60.79%), H (3.49%), N (18.66%), S (8.54%); Found: C (61.0%), H (3.6%), N (18.5%), S (8.6%).

### 3.2. Antimicrobial Screening

The newly synthesized heterocyclic compounds were tested for their antimicrobial activity against the following microorganisms: (a) Gram-negative: *Escherichia coli* and *Pseudomonas putide*; (b) Gram-positive: *Bacillus subtilis* and *Streptococcus lactis*; (c) Fungi: *Aspergillus niger* and *Penicillium sp.*; (d) Yeast: *Candida albicans. Media:* Three types of specific media were used in this study: 

*Medium 1:* For bacteria (Nutrient Medium), consisting of (g/L distilled water): peptone, 5 and meat extract, 3. pH was adjusted to 7.0. 

*Medium 2:* For fungi (Potato Dextrose Medium), consisting of (g/L distilled water): Infusion from potatoes, 4 and D(+)glucose, 20. pH was adjusted to 5.5. 

*Medium 3:* For yeast (Universal Medium), consisting of (g/L distilled water): yeast extract, 3; malt extract, 3; peptone, 5 and glucose, 10. pH was adjusted to 5.5. 

For solid media, 2% agar was added. All media were sterilized at 121 °C for 20 min. 

#### 3.2.1. Procedure (Filter paper diffusion method) [25]

Proper concentrations of microbial suspensions were prepared from 1 (for bacteria) to 3 (for yeast and fungi)-day-old liquid stock cultures incubated on a rotary shaker (100 rpm). In the case of fungi, five sterile glass beads were added to each culture flask. The mycelia were then subdivided by mechanical stirring at speed No. 1 for 30 min. Turbidity of microorganisms was adjusted with a spectrophotometer at 350 nm to give an optical density of 1.0. Appropriate agar plates were aseptically surface inoculated uniformly by a standard volume (ca. 1 mL) of the microbial broth culture of the tested microorganism, namely *E. coli*, *P. putida*, *B. subtilis*, *S. lactis*, *A. niger*, *Penicillium sp.* and *C. albicans.*

Whatman No. 3 filter paper discs of 10 mm diameter were sterilized by autoclaving for 15 min at 121 °C. Test compounds were dissolved in 80% ethyl alcohol to give final concentration of 5 μg/mL. The sterile discs were impregnated with the test compounds (5 μg/disc). After the impregnated discs have been air dried, they were placed on the agar surface previously seeded with the organism to be tested. Discs were gently pressed with forceps to insure thorough contact with the media. Three discs were arranged per dish, suitably spaced apart, *i.e.* the discs should be separated by a distance that is equal to or slightly greater than the sum of the diameters of inhibition produced by each disc alone. Each test compound was conducted in triplicate. Plates were kept in the refrigerator at 5 °C for 1 h to permit good diffusion before transferring them to an incubator at 37 °C for 24 h for bacteria and at 30 °C for 72 h for yeast and fungi.

### 3.3. Anti-Oxidant Screening

#### 3.3.1 Assay for erythrocyte hemolysis

Blood was obtained from rats by cardiac puncture and collected in heparinized tubes. Erythrocytes were separated from plasma and the buffy coat and washed three times with 10 volumes of 0.15 M NaCl. During the last washing, the erythrocytes were centrifuged at 2,500 rpm for 10 min to obtain a constantly packed cell preparation. Erythrocyte hemolysis was mediated by peroxyl radicals in this assay system [26]. A 10% suspension of erythrocytes in pH 7.4 phosphate-buffered saline (PBS) was added to the same volume of 200 mM 2,2'-azobis(2-amidinopropane)dihydrochloride (AAPH) solution (in PBS) containing samples to be tested at different concentrations. The reaction mixture was shaken gently while being incubated at 37 °C for ~h. The reaction mixture was then removed, diluted with eight volumes of PBS and centrifuged at 2,500 rpm for 10 min. The absorbance A of the supernatant was read at 540 nm. Similarly, the reaction mixture was treated with eight volumes of distilled water to achieve complete hemolysis, and the absorbance B of the supernatant obtained after centrifugation was measured at 540 nm. The percentage hemolysis was calculated by equation (1 − A/B) × 100%. The data were expressed as mean standard deviation. L-Ascorbic was used as a positive control.

#### 3.3.2. Anti-oxidant activity screening assay-ABTS method

For each of the investigated compounds, 2 mL of ABTS [2,2'-azino-bis(3-ethylbenzthiazoline-6-sulphonic acid)] solution (60 mM) was added to MnO_2_ suspension, all prepared in phosphate buffer (pH 7, 0.1 M). The mixture was shaken, centrifuged, filtered to remove excess MnO_2_, and the absorbance (A_control_) of the resulting green-blue solution (ABTS radical solution) was adjusted at ca. 0.5 at λ 734 nm. Then, 50 μL of (2 mM) solution of the test compound in spectroscopic grade MeOH/ phosphate buffer (1:1) was added. The absorbance (A_test_) was measured and the reduction in color intensity was expressed as % inhibition. The % inhibition for each compound is calculated from the following equation [27]:

$$\%\;Inhibition=\frac{Acontrol-Asample}{Acontrol}\times 100$$

Ascorbic acid (vitamin C) was used as standard anti-oxidant (positive control). Blank sample was run without ABTS and using MeOH/phosphate buffer (1:1) instead of sample. Negative control sample was run with MeOH/phosphate buffer (1:1) instead of tested compound.

#### 3.3.3. Bleomycin-dependent DNA damage

The assay was done according to Aeschlach *et al.* [28] with minor modifications. The reaction mixture (0.5 mL) contained DNA (0.5 mg/mL), bleomycin sulfate (0.05 mg/mL), MgCl_2_ (5 mM), FeCl_3_ (50 μM) and samples to be tested at different concentrations. L-Ascorbic acid was used as a positive control. The mixture was incubated at 37 °C for 1 h. The reaction was terminated by addition of 0.05 mL EDTA (0.1 M). The color was developed by adding 0.5 mL thiobarbituric acid (TBA) (1%, w/v) and 0.5 mL HCl (25%, v/v) followed by heating at 80 °C for 10 min. After centrifugation, the extent of DNA damage was measured by increase in absorbance at 532 nm.

## 4. Conclusions 

New spiro-compounds have been synthesized using both traditional methods and microwave-assisted conditions. The latter methods proved much more efficient in reducing reaction times as well as increasing the overall yield of the reactions. The newly synthesized compounds were tested for their antimicrobial and antioxidant activities. Some compounds showed moderate or weak antimicrobial activity, whereas compounds **7** and **8** showed promising antioxidant activity.

## Figures and Tables

**Table 1:** Antimicrobial activities of the newly synthesized compounds.

Comp.No.	Inhibition zone (mm)						
	Gram-negative		Gram-positive		Fungi	Yeast	
	*E. coli*	*P. putida*	*B. subtilis*	*S. lactis*	*A. niger*	*P. sp.*	*C. albicans*
**4a**	8	4	4	6	3	2	0
**4b**	0	0	0	0	0	0	0
**5a**	5	3	5	5	4	3	0
**5b**	10	9	10	8	6	5	0
**6a**	0	0	0	0	0	0	0
**6b**	0	0	0	0	0	0	0
**7**	0	0	0	0	0	0	0
**8**	10	8	8	7	8	5	0
**Chloram-phenicol®**	22	21	18	19	20	12	0
**Ampicilin®**	24	20	19	22	24	14	14

*E. coli* = *Escherichia coli*; *P. putida* = *Pseudomonas putida*; *B. subtilis* = *Bacillus subtilis*; *S. lactis* = *Streptococcus lactis*; *A. niger* = *Aspergillus niger*; *P. sp.* = *Penicillium sp*; *C. albicans* = *Candida albicans*. The sensitivity of microorganisms to the tested compounds is identified in the following manner *: Highly sensitive = Inhibition zone 15-20 mm; Moderately sensitive = Inhibition zone: 10-15 mm; Slightly sensitive = Inhibition zone: 5-10 mm; Not sensitive = Inhibition zone: 0 mm;* Each result represents the average of triplicate readings.

**Table 2:** Anti-oxidant assays by erythrocyte hemolysis (A/B × 100).

Compounds	Absorbance of samples (A)	Hemolysis (%)
**Complete hemolysis with distilled water (B)**	0.660	–
**Ascorbic acid**	0.026	3.93
**4a**	0.048	7.27
**4b**	0.052	7.87
**5a**	0.213	32.37
**5b**	0.187	28.33
**6a**	0.062	9.39
**6b**	0.068	10.30
**7**	0.030	4.54
**8**	0.033	5.00

**Table 3:** Anti-oxidant assays by ABTS method [Abs. (control) − Abs. (test)/Abs. (control) × 100].

Compounds	Absorbance of samples	Inhibition (%)
**ABTS control**	0.54	0
**Ascorbic acid**	0.06	88.9
**4a**	0.18	66.7
**4b**	0.20	63.0
**5a**	0.40	25.9
**5b**	0.42	22.2
**6a**	0.30	44.4
**6b**	0.32	40.7
**7**	0.11	79.6
**8**	0.10	81.5

**Table 4:** Assays for bleomycin-dependent DNA damage.

Compound	Absorbance of samples
**Ascorbic acid**	0.020
**4a**	0.038
**4b**	0.040
**7**	0.024
**8**	0.026
